# Respiratory tract infections in Norwegian primary care 2006–2015: a registry-based study

**DOI:** 10.1080/02813432.2022.2069711

**Published:** 2022-04-29

**Authors:** Leo Larsen, Knut-Arne Wensaas, Knut Erik Emberland, Guri Rortveit

**Affiliations:** a Department of Global Public Health and Primary Care, Section for General Practice, University of Bergen, Bergen, Norway; b Department of Health, Research Unit for General Practice, NORCE Norwegian Research Centre AS, Bergen, Norway

**Keywords:** Data analysis, epidemiology, general practice, health services research, primary health care, respiratory tract infections

## Abstract

**Objective:**

Examine characteristics and time trends of respiratory tract infection (RTI) consultations in Norwegian primary care and compare consultations in daytime general practice and out-of-hours (OOH) services.

**Design:**

Registry-based study using reimbursement claims data.

**Setting:**

All in-person primary care consultations during 2006–2015.

**Patients:**

All patients visiting primary care during the study period.

**Main outcome measures:**

The main outcome variable was RTI consultations. Differences regarding service type (general practice or OOH services) and changes over time were investigated. We report associations with patient age and sex, season, point-of-care C-reactive protein (CRP) test use, and sickness certificate issuing.

**Results:**

RTI consultations (*n* = 16 304 777) represented 11.6% of all consultations (*N* = 140 199 637) in primary care over the ten-year period. The annual number of RTI consultations per 1000 inhabitants decreased from 335 to 314, while the number of consultations for any reason increased. Of RTI consultations, 83.2% occurred in general practice. OOH services had a higher proportion of RTI consultations (21.4%) compared with general practice (10.6%). Young children (0–4 years) represented 18.9% of all patients in RTI consultations. CRP testing was used in 56.2% of RTI consultations, and use increased over time. Sickness certificates were issued in 31.9% of RTI consultations with patients of working age (20–67 years).

**Conclusion:**

Most RTI consultations occurred in general practice, although the proportion was higher in OOH services. Laboratory testing and/or issuing of sickness certificates were part of most consultations. This could be an important reason for seeking health care.

**Key Points**
- Patients with a respiratory tract infection (RTI) are mostly managed in primary care, where they represent much of the workload.
- Most consultations for RTIs took place in daytime general practice, but out-of-hours services had a higher proportion of RTI consultations.
- RTIs were the dominating reason for encounter among young children both in out-of-hours services and daytime general practice.
- CRP tests were used in over half of RTI consultations, and their use expanded over time.

## Introduction

Respiratory tract infections (RTIs) affect a great number of patients every year. RTIs account for a large proportion of short-term absence from work and has a significant impact on sickness related societal costs [1]. In all Nordic countries, RTIs have historically been reported as one of the most common reason for consultations in primary care [2,3]. Also, in the Netherlands, a country with a similar health care system to Norway, a study from 2017 reported acute upper RTIs to be the most common reason for children to visit primary care [4]. A recent study of visits due to infections in Swedish primary care found that RTIs were the most common reason during the study period 2006–2014 [5].

Most patients seeking health care for acute RTIs are treated in primary care without further referral. Norway has a universal health care system, and general practitioners (GPs) act as gatekeepers to secondary care. Outside regular office hours emergency medical inquiries are managed by the out-of-hours (OOH) services, mainly staffed by GPs on duty. The impact of the differences between daytime general practice and OOH services on the management of RTIs has not been thoroughly investigated.

Workload is increasing in general practice, and both increase in consultation rates and time constraint during the consultations are risk factors for unnecessary antimicrobial prescription [6,7]. In Norway, more than 80% of antimicrobials are prescribed in primary care, with RTIs as the main reason for prescription [8]. Documenting the incidence of RTIs in primary care in Norway will provide a necessary overview for planning and improving quality of health services, as well as addressing inappropriate use of antibiotics.

With these factors in mind, studies that chart the use of primary health care for RTIs are of importance. The aims of this study were to determine characteristics and time trends of consultations for RTIs in Norwegian primary care, and to compare RTI consultations in general practice and OOH services.

## Methods

All citizens in Norway are entitled to a GP through a registered list system, and 99.8% of the population is registered to this service [9].

The municipalities are responsible for primary health care services, which also includes OOH services. The OOH services are normally provided by municipal emergency rooms staffed by local GPs in rotation.

### Study design

We obtained reimbursement claims data from the National registry for control and reimbursements of health care interventions in primary care (KUHR).

The current study uses data from all consultations by encounter in the period 2006–2015, and variables in the data set include diagnostic codes according to the International Classification of Primary Care version 2 (ICPC-2), age and sex of patients, quarter and year of consultation, as well as codes for the reimbursed procedures C-reactive protein (CRP) point-of-care tests and issuing of sickness certificates.

### Outcome

The main outcome in this study was face-to-face consultations with RTI diagnoses. Included ICPC-2 codes were chosen based on previous literature in the field and clinical experience of the research group [10–12]. We based our definition of RTIs on the one used by Statistics Norway [13] but altered the inclusion slightly by adding codes for acute otitis externa and abnormal sputum/phlegm. We defined ‘RTI consultation’ as a consultation with one or more of the following codes (grouped by anatomical region): Ears (H70, H71, H72, H74), sinuses (R09, R75), throat (R21, R72, R76), upper respiratory tract (R05, R71, R74, R77, R80, R83), lower respiratory tract (R25, R78, R81, R82).

Differentiating consultations in general practice from OOH services was done based on predefined categories in the registry. We categorized age into 10-year groups, except for the youngest (0–4 years) and oldest (≥85 years). We defined the first (January–March) and fourth quarter (October–December) of the year as the winter season, and the second (April–June) and third (July–September) quarters as the summer season. Only patients aged 20–67 years were included in the analyses of sickness certificates.

### Analysis

Data analyses were conducted in Stata/SE 16.0 for Windows 10 and Microsoft Office 365 Excel Version 2001. Descriptive analyses included frequency distributions and contingency tables analyses. We investigated possible associations between RTI consultations and patient age, sex, quarter and year of consultation, CRP test use, and issuing of sickness certificates, respectively, by using contingency tables analysis. Missing values were omitted from analyses by listwise deletion. Data presented as annual numbers of consultations per 1000 inhabitants were based on the Norwegian population as of January 1 each year (available from Statistics Norway’s online Statbank) [14].

The study was approved by the Regional Committee for Medical and Health Research Ethics, approval ID 2018/1541.

## Results

### Study population and administrative characteristics

During the ten-year study period, there were a total of 140 199 637 consultations in Norwegian primary care, of which 16 304 777 (11.6%) were for RTIs (Table 1). The number of RTI consultations per 1000 inhabitants decreased from 335 in 2006 to 314 in 2015, whereas consultations for any reason increased from 2650 to 2940 per 1000 inhabitants (Table 2). Correspondingly, the proportion of consultations for RTIs decreased from 12.6% to 10.7%.

**Table 1.** Consultations for any diagnosis and for respiratory tract infection (RTI) diagnoses in Norwegian daytime general practice (DGP) and out-of-hours (OOH) services (2006–2015).

	Consultations for any diagnosis						Respiratory tract infection consultations					
	GP + OOH		GP		OOH		OOH + GP		GP		OOH	
	*n*	%	*n*	%	*n*	%	*n*	%	*n*	%	*n*	%
Total	140 199 637	100^a^	127 389 382	90.9^a^	12 810 255	9.1^a^	16 304 777	100^a^	13 560 052	83.2^a^	2 744 725	16.8^a^
Sex												
Male	59 049 592	42.1	52 958 422	41.6	6 091 170	47.5	7 157 919	43.9	5 872 171	43.3	1 285 748	46.8
Female	81 149 996	57.9	74 430 921	58.4	6 719 075	52.5	9 146 852	56.1	7 687 877	56.7	1 458 975	53.2
Missing	49		39		10		6		4		2	
Age group												
0–4	7 469 970	5.3	5 699 054	4.5	1 770 916	13.8	3 088 545	18.9	2 332 538	17.2	756 007	27.5
5–14	8 044 813	5.7	6 632 863	5.2	1 411 950	11.0	1 844 873	11.3	1 470 516	10.8	374 357	13.6
15–24	12 912 593	9.2	10 962 960	8.6	1 949 633	15.2	1 984 711	12.2	1 598 452	11.8	386 259	14.1
25–34	17 936 332	12.8	16 204 321	12.7	1 732 011	13.5	2 063 879	12.7	1 733 593	12.8	330 286	12.0
35–44	19 466 283	13.9	17 906 604	14.1	1 559 679	12.2	2 142 618	13.1	1 847 838	13.6	294 780	10.7
45–54	19 361 034	13.8	18 077 251	14.2	1 283 783	10.0	1 693 016	10.4	1 495 091	11.0	197 925	7.2
55–64	19 962 550	14.2	18 834 073	14.8	1 128 477	8.8	1 597 455	9.8	1 426 344	10.5	171 111	6.2
65–74	16 469 661	11.7	15 611 736	12.3	857 925	6.7	1 023 910	6.3	913 590	6.7	110 320	4.0
75–84	13 145 913	9.4	12 430 614	9.8	715 299	5.6	631 421	3.9	549 842	4.1	81 579	3.0
85–	5 430 438	3.9	5 029 866	3.9	400 572	3.1	234 343	1.4	192 244	1.4	42 099	1.5
Missing	50		40		10		6		4		2	
Season												
January–March	36 239 587	25.8	33 101 417	26.0	3 138 170	24.5	5 370 444	32.9	4 509 655	33.3	860 789	31.4
April–June	34 630 198	24.7	31 357 589	24.6	3 272 609	25.5	3 377 505	20.7	2 764 427	20.4	613 078	22.3
July–September	32 226 854	23.0	29 069 703	22.8	3 157 151	24.6	2 978 600	18.3	2 473 671	18.2	504 929	18.4
October–December	37 102 998	26.5	33860 673	26.6	3 242 325	25.3	4 578 228	28.1	3 812 299	28.1	765 929	27.9
CRP												
No	118 535 702	84.5	109 854 835	86.2	8680 867	67.8	7 145 068	43.8	6 102 459	45.0	1 042 609	38.0
Yes	21 663 935	15.5	17 534 547	13.8	4 129 388	32.2	9 159 709	56.2	7 457 593	55.0	1 702 116	62.0
Sickness certificate^b^												
No	68 938 272	76.9	62 522 490	75.8	6 415 782	90.7	6 021 680	68.1	5 007 455	65.8	1 014 225	82.6
Yes	20 658 152	23.1	19 997 369	24.2	660 783	9.3	2 814 832	31.9	2 601 206	34.2	213 626	17.4
Total	89 596 424	100	82 519 859	100	7 076 565	100	8 836 512	100	7 608 661	100	1 227 851	100

Distribution within sex, age, season, point-of-care CRP, and sickness certificate is given by column if not otherwise stated.

^a^Percentage of service type (DGP and OOH services) for consultations ‘any diagnosis’ and ‘respiratory tract infection consultations’ respectively.

^b^Analyses of sickness certificate are restricted to patients aged 20–67 years.

**Table 2.** Annual consultations per 1000 inhabitants for any diagnoses and for respiratory tract infection (RTI) diagnoses, and percentage of RTI consultations in relation to the total, in Norwegian daytime general practice (DGP) and out-of-hours (OOH) services (2006–2015).

	GP + OOH			GP			OOH		
Year	Any diagnosis	RTI	%	Any diagnosis	RTI	%	Any diagnosis	RTI	%
2006	2 650	335	12.6	2 397	275	11.5	253	60	23.9
2007	2 686	343	12.8	2 432	283	11.6	253	60	23.8
2008	2 788	336	12.1	2 520	275	10.9	268	62	23.0
2009	2 805	341	12.2	2 539	281	11.1	266	61	22.9
2010	2 887	326	11.3	2 620	268	10.2	267	58	21.7
2011	2 973	353	11.9	2 704	294	10.9	269	59	22.1
2012	2 983	359	12.0	2 715	300	11.0	268	59	22.1
2013	2 939	322	10.9	2 678	271	10.1	261	51	19.5
2014	2 957	304	10.3	2 698	258	9.5	259	47	18.0
2015	2 940	314	10.7	2 687	269	10.0	253	46	18.1
Total	2 864	333	11.6	2 603	277	10.6	262	56	21.4

The vast majority of consultations took place in general practice compared to OOH services: 90.9% of consultations for any reason and 83.2% for RTIs (Table 1). In general practice, 277 of 2 603 consultations per 1000 inhabitants (10.6%) were for RTIs. In OOH services, 56 of 262 consultations per 1000 inhabitants (21.4%) were for RTIs (Table 2).

More than one RTI diagnosis code was registered in 464 169 (2.8%) of the RTI consultations. The most frequent RTI diagnosis code was ‘R74 acute upper respiratory infection’, accounting for 27% of consultations for RTIs. There were no major differences between the sexes in the distribution of the RTI diagnoses (data not shown).

### Seasonal variation

**Figure 1.**
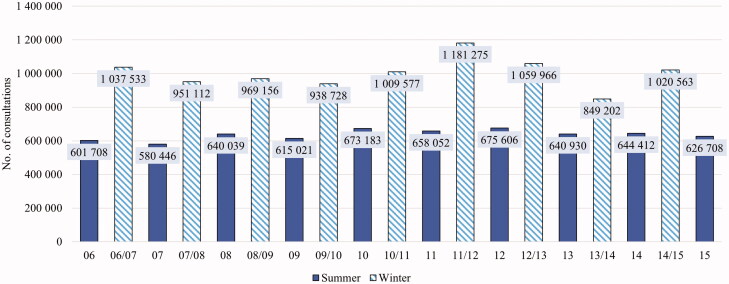
Seasonal respiratory tract infection (RTI) consultations in Norway (2006–2015).

In the ten-year period, 61% of all RTI consultations took place during the winter months, compared to 52% of consultations for any reason. This trend of seasonal variation was observed every year, but the absolute number of RTI consultations changed more between winter seasons than it did between summers (Figure 1). The difference between the number of RTI consultation in the winter and summer seasons was greatest for patients aged 0–14, where 67% of all RTI consultations took place in the winter season. For patients aged 15–24, there was almost no seasonal difference for RTI consultations (53% in winter).

### Associations with patient sex and age

There was an overall majority of female patients in consultations both for any reason and for RTIs (Table 1), and this difference was larger in general practice than in OOH services. In the lowest age group (0–4 years), there was a majority of male patients in consultations for any reason (54.2%) and for RTIs (54.8%). In the age group 5–14, there was essentially no difference between sexes (data not shown). For all other age groups, there was a majority of female patients in consultations for any reason (59.2%) and for RTIs (60.0%).

Age was strongly associated with the number of RTI consultations (Table 1). In the lowest age group, RTIs accounted for 41.3% of all consultations, with little difference between general practice (42.7%) and OOH services (40.9%). This proportion dropped consistently with age to 4.3% of RTI consultations in the highest age group, albeit with greater differences between general practice (3.8%) and OOH services (10.5%) in this age group. The lowest age group including only five birth cohorts (0–4 years) also had the highest total number of RTI consultations, followed by patients aged 35–44 (Table 1).

### Point-of-care CRP test use

**Figure 2.**
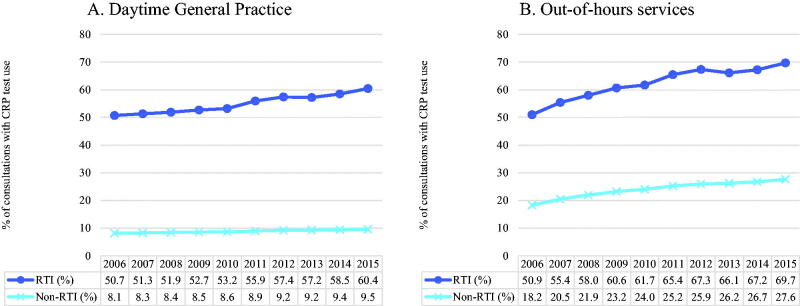
(A and B) Annual percentage of consultations with point-of-care C-reactive protein (CRP) test use, by respiratory tract infection (RTI) status, in Norwegian daytime +general practice and out-of-hours services (2006–2015).

CRP tests were used in 56.2% of the RTI consultations and in 8.9% of the non-RTI consultations, with no major seasonal variation (data not shown). From 2006 to 2015, the proportion of RTI consultations where a CRP test was used increased from 50.7% to 60.4% in general practice, and from 50.9% to 69.7% in OOH services. For consultations for other reasons, CRP test use increased only from 8.1% to 9.5% in general practice, while it increased from 18.2% to 27.6% in OOH services (Figure 2).

The use of CRP tests for RTIs did not differ between sexes. In both OOH services and in general practice, the use of CRP tests increased with increasing patient age over 45 years, whereas for younger patients there was little difference between age groups (data not shown).

### Sickness certificate issuing

**Figure 3.**
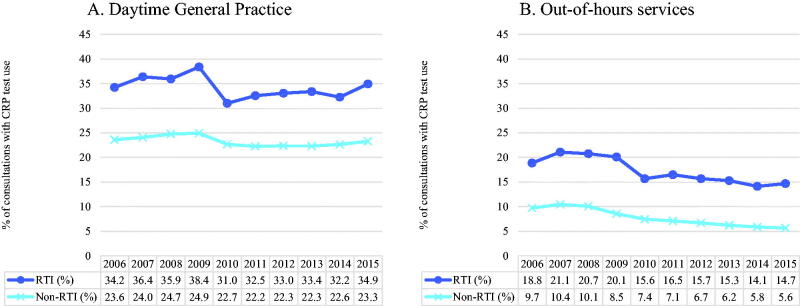
(A and B) Annual percentage of consultations with sickness certificate issuing, by respiratory tract infection (RTI) status, in Norwegian daytime general practice and out-of-hours services (2006–2015).

A sickness certificate was issued in 31.9% of the RTI consultations in the age group 20–67 years, with the proportion in general practice (34.2%) being roughly twice that of OOH services (17.4%). In daytime general practice, the proportion of consultations with sickness certificates issued did not change much over the ten-year period (34.2% to 34.9% for RTI, and 23.6% to 23.3% for non-RTI consultations) (Figure 3(A)). In contrast, the proportion of OOH consultations with sickness certificates decreased gradually, by 4.1 percentage points, in both RTI (18.8% to 14.7%) and non-RTI (9.7% to 5.6%) consultations (Figure 3(B)). The proportion of RTI consultations with a sickness certificate issued was highest among young patients. The age group 25–34 years was the one with the highest proportion of RTI consultations with sickness certificates issued in general practice (40.6%), and patients aged 20–24 years had the highest proportion in OOH services (21.9%). Older patient groups had subsequently lower proportions of RTI consultations with sickness certificates, with the lowest among patients aged 65–67 years (10.0% in general practice and 3.9% in OOH services).

## Discussion

### Summary

Most consultations for RTIs took place in daytime general practice in the study period, although the proportion of RTI consultations among all consultations in OOH services was higher. RTIs were the dominating reason for encounter among young children. The number of RTI consultations was stable over the study period, while the number of consultations for other reasons increased. CRP testing was used in more than half of RTI consultations, and the use increased both in daytime general practice and in OOH services during the study period.

### Strengths and limitations

To our knowledge, this is the first study of RTI consultations in Norwegian primary care with complete national data over a full 10-year period. The inclusion of all consultations in Norwegian primary care in the study period is a major strength of this study. Consequently, the external validity is high because the sample population is equal to the target population. An updated data set with more recent data would have further increased the value of this study but were not available as access to registry data in Norway requires extensive and resource-demanding procedures.

Diagnostic codes on reimbursement claims are reasonably accurate in this setting, according to studies from Norway and other countries [15–17]. Home visits and telephone consultations were not included in the data material, implying that the current study may not give the full picture of the burden of RTIs in Norwegian primary care. However, we do not expect inclusion of telephone consultations and home visits to dramatically change the overall findings, as most RTIs traditionally have been managed by regular consultations by encounter. Also, diagnoses from other than face-to-face consultations are shown to be less accurate [17].

Drop-in GP services outside the national health system were not largely established in Norway during the study period, and as such are not expected to significantly impact the findings in the study.

The entity of this study was consultations for RTIs, rather than infection episodes. This approach provides insight about the impact on health care services but is less accurate for collecting information on the incidence or prevalence of RTIs in primary care, since patients may consult several times for the same infection. Compared to a study of individual infection episodes, issuing of sickness certificates may appear underestimated. Also, rates of CRP testing may appear differently when investigating episodes rather than stand-alone consultations.

It is important to note that the findings in our study is inextricably linked to the organization of the Norwegian health care system, and one should take care before extrapolating results from this study to countries with differing organization of health care. However, we believe that the main features are generalizable to other high-income countries with a strong primary care system.

### Comparison with previous literature

Our findings that RTIs are a common reason for visiting primary care, as well as acute upper RTI being the most common RTI diagnosis, is in line with previous studies from other countries [3,18,19]. A systematic review from 2018 concluded that, globally, RTIs are the most common reason for seeking primary care [18].

Compared to the study from Swedish primary care [5], we found slightly higher numbers of consultations for RTIs per 1000 inhabitants in Norway. This may not necessarily reflect real differences in RTI incidence, but rather differences in study designs and methods, health care seeking behavior and/or organization of health care services, between the two countries.

The annual number of RTI consultations did not change much over time, despite the 11.3% increase in the population during the study period. We cannot say whether this reflects a relative decrease in RTIs in the community or changes in health care seeking behavior. The annual number of consultations for any reason increased by 24% over the period, which indicates an increased use of primary care services for other reasons.

As expected, a higher number of RTI consultations took place during the winter seasons compared to the summer. The 2009 H1N1 influenza pandemic was included in the study, and we found that during the whole study period, the winter of 2009/2010 had the second lowest number of consultations for RTIs (Figure 1). A previous study found that the number of diagnoses for influenza-like-illness in Norway increased 3.3-fold for the 2009/2010 winter season compared to the previous winter [20]. A possible explanation is that the increased measures to limit the spread of infection had an impact on the incidence of all infections, or on health care seeking behavior for self-limiting infections. Another explanation could be that an increased awareness of the pandemic by patients and physicians shifted diagnostic focus from RTIs generally to influenza specifically. This finding should be kept in mind when trying to evaluate the workload for health care services during epidemics.

The season with the highest number of RTI consultations was the winter 2011/2012. This coincides with a large epidemic of Mycoplasma pneumoniae [21]. We did not perform any diagnosis-level analyses for this season but find it likely that the epidemic contributed to the high prevalence of RTI diagnoses.

The youngest patients dominate RTI consultations in both general practice and OOH services. This finding is in line with previous literature [4]. Furthermore, young adults also had high numbers of consultations for RTIs. One explanation for this might be that parents are infected by their children.

The proportion of RTI consultations in OOH services was higher than in general practice for all age groups, and especially so for the oldest. A plausible explanation is that RTIs when affecting the older may be more acute and severe, and therefore more often in need of emergency medical aid.

Antibiotic usage is reported to have risen from 2005 to 2012 and has fallen since [8], in contrast with stable numbers of annual RTI consultations.

CRP point-of-care tests are readily available in Norwegian primary care, and during the study period, their use increased both in daytime general practice and in OOH services, to 60 and 70% of RTI consultations, respectively. The observed increase can hardly be explained by a corresponding increase in more severe RTIs. Evidence shows that CRP testing reduce antibiotic prescribing for patients with acute RTIs and acute exacerbations of chronic obstructive pulmonary disease in primary care [22,23]. However, there is a concern that CRP testing might also have negative effects, for instance on costs of management or re-consultation, but there is limited measurement of these outcomes [22]. A study from OOH services in Norway where children with RTIs were randomized to either mandatory CRP testing or testing at the discretion of the physician found no effect on antibiotic prescription or hospital referrals [24]. There is a risk that a too liberal use of CRP testing will reduce self-care and increase health care seeking, and secondarily lead to increased prescription of antibiotics. In a study on the consequences of a change in school absence policy in Norway in 2016 the number of consultations among 16- to 18-year-olds increased by 30%, and the use of antibiotics in this group increased by 26%. This could not be explained by more serious infections, as there was no such increase among 15-year-olds, who were not affected by the change in policy [7].

Issuing of sickness certificates decreased in OOH services over the study period. There was a notable drop in consultations with sickness certificates between 2009 and 2010 for RTI consultations, less so for non-RTIs. Although interesting, we do not have an explanation for this finding. Sickness certificates being issued in higher proportions for younger patients suggests different reasons for visiting the health care services between age groups. A hypothesis that may need further investigation is that younger patients visit health care services for sickness certificates and older patients visit for treatment. The observation that sickness certificates were issued in RTI consultations twice as often in daytime general practice than in the OOH services is in line with the GPs’ responsibility for follow-up of patients.

### Implications

Our study provides important background information about RTIs in primary care between 2006–2015. The study may serve as a basis of comparison regarding RTI consultations in primary care services in Norway and other countries with a comparable primary care system.

## Conclusion

We found large differences between general practice and OOH services with regards to RTI consultations. We also discovered clear time trends in CRP test use and sickness certificate issuing. While RTI consultations seemed to vary greatly with age group, we observed no major sex differences.

## References

[1] Putri WCWS, Muscatello DJ, Stockwell MS, et al. Economic burden of seasonal influenza in the United States. Vaccine. 2018;36(27):3960–3966. 29801998 10.1016/j.vaccine.2018.05.057

[2] Grimsmo A, Hagman E, Falko E, et al. Patients, diagnoses and processes in general practice in the nordic countries. An attempt to make data from computerised medical records available for comparable statistics. Scand J Prim Health Care. 2001;19:76–82. 11482418 10.1080/028134301750235277

[3] Wandell P, Carlsson AC, Wettermark B, et al. Most common diseases diagnosed in primary care in Stockholm, Sweden, in 2011. Fam Pract. 2013;30(5):506–513. 23825186 10.1093/fampra/cmt033

[4] Dekker ARJ, Verheij TJM, van der Velden AW. Antibiotic management of children with infectious diseases in dutch primary care. Fam Pract. 2017;34(2):169–174. 28122841 10.1093/fampra/cmw125

[5] Cronberg O, Tyrstrup M, Ekblom K, et al. Diagnosis-linked antibiotic prescribing in swedish primary care – a comparison between in-hours and out-of-hours. BMC Infect Dis. 2020;20(1):11. 32819280 10.1186/s12879-020-05334-7 PMC7441551

[6] Hobbs FDR, Bankhead C, Mukhtar T, National Institute for Health Research School for Primary Care Research, et al. Clinical workload in UK primary care: a retrospective analysis of 100 million consultations in England, 2007–14. Lancet. 2016;387(10035):2323–2330. 27059888 10.1016/S0140-6736(16)00620-6 PMC4899422

[7] Bakken IJ, Wensaas K-A, Furu K, et al. General practice consultations and use of prescription drugs after changes to school absence policy. Tidsskr nor Laegeforen. 2017;137(16)1178-1184. 10.4045/tidsskr.17.0427 28871761

[8] NORM/NORM-VET 2018. Usage of Antimicrobial Agents and Occurrence of Antimicrobial Resistance in Norway [Internet]. Tromsø/Oslo: 2019 [cited 2020 Aug 26]. Available from: https://www.vetinst.no/en/surveillance-programmes/norm-norm-vet-report/_/attachment/download/375a14dd-f414-4ba3-9f89-2a3efe5fd79f:cac1f2c419e473923334222ca1a0c6ad5a4ac957/NORM%20NORM-VET%202018.pdf.

[9] Gaardsrud PØ. Styringsdata for fastlegeordningen, 4. kvartal2019. [Internet]. Helsedirektoratet; [cited 2021 Jan 22]. Available from: https://www.helsedirektoratet.no/statistikk/fastlegestatistikk/Hovedtallsrapport%20fastlegeordningen%20landstall%202019-4%20(002).pdf.

[10] Gjelstad S, Dalen I, Lindbaek M. GPs' antibiotic prescription patterns for respiratory tract infections-still room for improvement. Scand J Prim Health Care. 2009;27(4):208–215. 19929185 10.3109/02813430903438718 PMC3413912

[11] Gjelstad S, Straand J, Dalen I, et al. Do general practitioners' consultation rates influence their prescribing patterns of antibiotics for acute respiratory tract infections? J Antimicrob Chemother. 2011;66(10):2425–2433. 21784782 10.1093/jac/dkr295

[12] Bakken IJ, Wensaas KA, Grøneng GM, et al. Upper secondary school leving celebrations and final exams – consultations in general practice and emergency care. Tidsskr nor Laegeforen. 2017;137(10):713–716. 28551969 10.4045/tidsskr.17.0158

[13] Allmennlegetjenesten [Internet]. Statistics Norway2020 [cited 2021 Feb 19];Available from: https://www.ssb.no/helse/helsetjenester/statistikk/allmennlegetjenesten.

[14] 05803: Population 1 January and population changes during the calendar year, by contents and year. Statbank Norway [Internet]. Statistics Norway. 2021 [cited 2021 Jan 29];Available from: https://www.ssb.no/en/statbank/sq/10053351.

[15] Linder JA, Bates DW, Williams DH, et al. Acute infections in primary care: Accuracy of electronic diagnoses and electronic antibiotic prescribing. J Am Med Inform Assoc. 2006;13(1):61–66. 16221947 10.1197/jamia.M1780 PMC1380198

[16] Cadieux G, Tamblyn R. Accuracy of physician billing claims for identifying acute respiratory infections in primary care. Health Serv Res. 2008;43(6):2223–2238. 18665858 10.1111/j.1475-6773.2008.00873.x PMC2614002

[17] Sporaland GL, Mouland G, Bratland B, et al. General practitioners’ use of ICPC diagnoses and their correspondence with patient record notes. Tidsskr nor Laegeforen. 2019;139(15)1468-1472. 10.4045/tidsskr.18.0440 31642635

[18] Finley CR, Chan DS, Garrison S, et al. What are the most common conditions in primary care? Systematic review. Can Fam Physician. 2018;64(11):832–840. 30429181 PMC6234945

[19] Adar T, Levkovich I, Castel OC, et al. Patient's utilization of primary care: a profile of clinical and administrative reasons for visits in Israel. J Prim Care Community Health. 2017;8(4):221–227. 29034793 10.1177/2150131917734473 PMC5932740

[20] Simonsen KA, Hunskaar S, Sandvik H, et al. Capacity and adaptations of general practice during an influenza pandemic. PLoS One. 2013;8(7):e69408. 23874960 10.1371/journal.pone.0069408 PMC3715475

[21] Blystad H, Ånestad G, Vestrheim DF, et al. Increased incidence of Mycoplasma pneumoniae infection in Norway. Euro Surveill 2012. 2011;17(5):12–14. 10.2807/ese.17.05.20074-en 22321136

[22] Tonkin‐Crine SK, Tan PS, van Hecke O, et al. Clinician‐targeted interventions to influence antibiotic prescribing behaviour for acute respiratory infections in primary care: an overview of systematic reviews. Cochrane Database Syst Rev. 2017;2017:CD012252. 10.1002/14651858.CD012252.pub2 PMC6483738 28881002

[23] Gillespie D, Butler CC, Bates J, et al. Associations with antibiotic prescribing for acute exacerbation of COPD in primary care: secondary analysis of a randomised controlled trial. Br J Gen Pract. 2021;71(705):e266–72–e272. 33657007 10.3399/BJGP.2020.0823 PMC8007268

[24] Rebnord IK, Sandvik H, Batman Mjelle A, et al. Out-of-hours antibiotic prescription after screening with C reactive protein: a randomised controlled study. BMJ Open. 2016;6(5):e011231. 10.1136/bmjopen-2016-011231 PMC4874126 27173814

