# Correction

**DOI:** 10.1080/02813432.2023.2250170

**Published:** 2023-09-04

**Article title:** “Respiratory tract infections in Norwegian primary care 2006-2015: A registry-based study”

**Authors**: Larsen, L., Wensaas, K.-R., Emberland, K. E., and Rortveit, G.

**Journal:** *Scandinavian Journal of Primary Health Care*

**Bibliometrics:** Volume 40, Number 2, pages 173–180

**DOI:** 10.1080/02813432.2022.2069711

When this article was first published online, Figure 3 of the *y*-axis was labelled “% of consultations with CRP test use” and should be labelled “% of consultations with sickness certificate issuing”. This has now been corrected with an updated Figure 3. 

**Figure 3.**
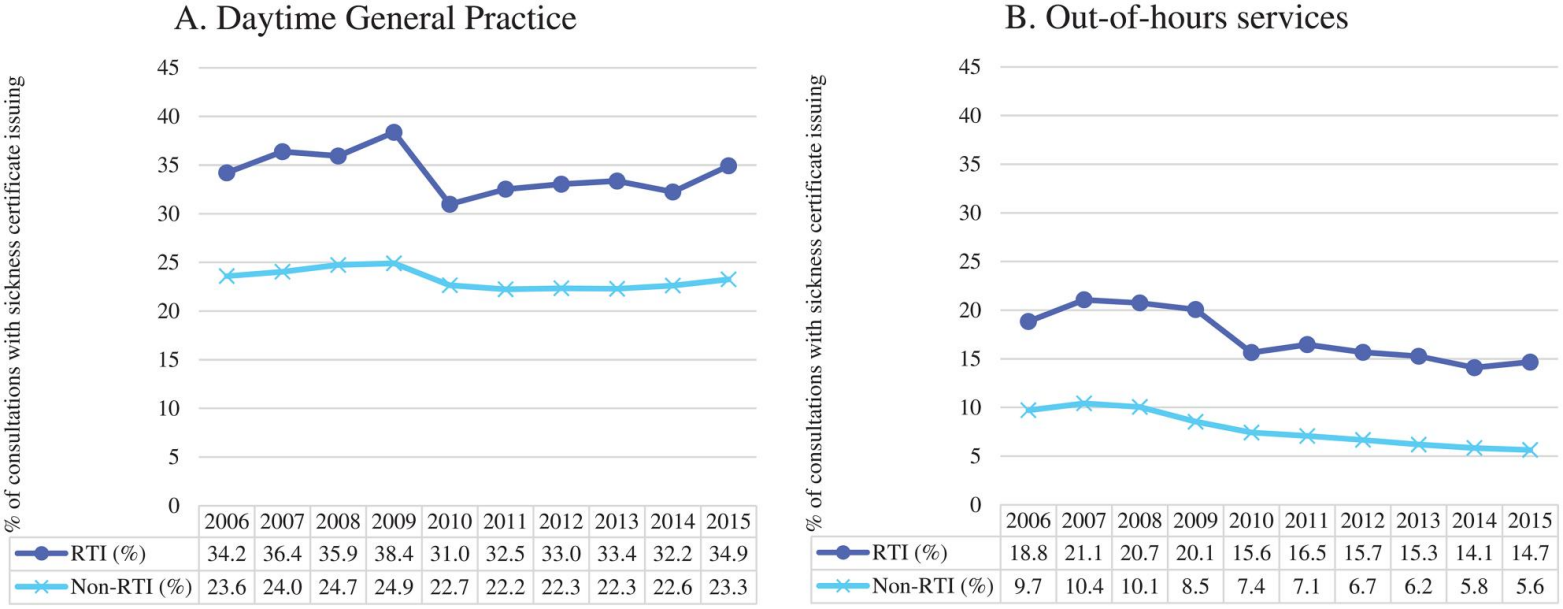
(A and B) Annual percentage of consultations with sickness certificate issuing, by respiratory tract infection (RTI) status, in Norwegian daytime general practice and out-of-hours services (2006–2015).

